# Baricitinib in chronic kidney disease: an exploratory analysis integrating network toxicology, molecular docking and pharmacovigilance

**DOI:** 10.3389/fmed.2025.1717739

**Published:** 2026-01-09

**Authors:** Rubin Zheng, Jing Lu, Miao Deng, Jiayi Lyu, Jinfen Han, Jiaxi Chen, Qin Wang, Ye Liu, Liangdong Yuan, Zhixun Bai

**Affiliations:** 1 Department of Nephrology, Qianxinan Affiliated Hospital of Zunyi Medical University, Xingyi, Guizhou, China; 2 Department of Nephrology, People’s Hospital of Qianxinan Prefecture, Xingyi, Guizhou, China; 3 Clinical College, Zunyi Medical University, Zunyi, Guizhou, China; 4 Department of Nursing, Southwest Guizhou Vocational and Technical College Nationalities, Xingyi, Guizhou, China; 5 Department of Nephrology, Affiliated Hospital of Jining Medical University, Jining, Shandong, China

**Keywords:** baricitinib, chronic kidney disease, molecular docking, network toxicology, pharmacovigilance

## Abstract

**Background:**

Chronic kidney disease (CKD) presents a major global health challenge due to ineffective therapies against progressive renal fibrosis. Baricitinib, a selective JAK1/JAK2 inhibitor, has anti-inflammatory and anti-fibrotic potential, yet its mechanistic basis and safety implications in CKD require further exploration.

**Methods:**

An integrated strategy was employed, combining network toxicology across multiple databases, protein-protein interaction network analysis and molecular docking. Real-world safety was evaluated by analyzing adverse event (AE) reports from FDA Adverse Event Reporting System (FAERS) (2018–2024), capturing safety data across all approved indications for baricitinib by calculating reporting odds ratios (RORs) and proportional reporting ratios (PRRs).

**Results:**

Predictive toxicology indicated potential respiratory and acute toxicity risks. Network analysis identified 229 shared targets; core hubs (AKT1, SRC, STAT3, EGFR, ESR1) showed high-affinity docking, suggesting potentially stronger theoretical binding affinity than JAK1. Pathway enrichment suggested potential suppression of JAK-STAT/MAPK and TGF-β/Smad3 pathways. FAERS analysis of 6,006 reports from its broader clinical use showed significantly elevated RORs for infections and thromboembolic events, alongside the absence of a disproportionate signal for renal AEs. This finding aligns with the mechanistic profile derived from intersecting baricitinib’s predicted targets with CKD-related genes, highlighting the need to systematically evaluate renal safety in prospective CKD trials.

**Conclusion:**

Baricitinib has computational and mechanistic potential to modulate key pathways in CKD. Pharmacovigilance data confirm risks of infection and thrombosis but show no disproportionate renal safety signal. These exploratory findings generate a testable hypothesis for its use in CKD, underscoring the necessity of prospective, renal-function-stratified trials.

## 1 Introduction

Chronic kidney disease (CKD) is a systemic condition marked by a continual decline in renal function and has become a major global public health concern due to its high prevalence and mortality rates. Recent epidemiological data indicate a worldwide CKD prevalence of 9.1–13.4% (stages 1–5), with complications like anemia and cardiovascular disease (CVD) significantly compromising patient outcomes and escalating healthcare costs (1–3). By 2019, 697 million individuals were living with CKD, and 1.427 million deaths were attributed to the disease, with population growth being the primary driver of case expansion (4). Projections indicate that by 2040, CKD will rank as the fifth leading cause of death worldwide, disproportionately affecting low− and middle−income countries because of limited access to therapies (5). Notably, patients experiencing >30% estimated glomerular filtration rate (eGFR) decline within 2 years face a 64% risk of progressing to end-stage renal disease (ESRD) and 50% mortality risk within 10 years (6).

Currently, sodium−glucose cotransporter−2 inhibitors (SGLT−2i) and renin−angiotensin−aldosterone system (RAAS) inhibitors serve as the primary therapeutic strategies for CKD, no treatments exist to reverse established renal interstitial fibrosis—a pivotal event in CKD progression involving myofibroblast activation, epithelial-mesenchymal transition (EMT), and crosstalk between inflammatory and metabolic pathways (7). Traditional single-target therapies against pro-inflammatory cytokines (e.g., IL-6, TNF-α) show limited efficacy due to their inability to address multi-pathway dysregulation (8, 9).

Baricitinib, an oral dual JAK1/2 inhibitor, has proven effective in rheumatoid arthritis (RA) by blocking IL−6/JAK/STAT signaling and reducing structural joint damage. Preclinical studies further show its ability to downregulate the TGF-β/Smad3 axis, reducing renal fibroblast-to-myofibroblast transition and offering a molecular rationale for antifibrotic effects (10, 11). This molecular rationale is corroborated by functional outcomes in key preclinical models, including attenuated renal fibrosis in the 5/6 nephrectomy (Nx) model (12), reduced inflammation and collagen deposition in unilateral ureteral obstruction (13), and improved albuminuria in diabetic kidney disease (DKD) models (14). However, these studies primarily address isolated pathways or phenotypic outcomes, leaving the integrated systems-level pharmacology of baricitinib largely uncharacterized. This encompasses its broader target engagement and associated safety implications within the complex milieu of CKD. However, its use in CKD is complicated by pharmacokinetic challenges: primarily metabolized by CYP3A4 with 75% renal excretion, clearance decreases by about 40% in moderate renal insufficiency (15). Real-world data highlight dose-dependent risks of adverse events (AEs), including elevated liver enzymes and deep vein thrombosis (DVT), necessitating close monitoring (16, 17).

Multi-dimensional safety assessments reveal complex effects: FDA Adverse Event Reporting System (FAERS) analysis showed a 1.89 odds ratio for metabolic acidosis, raising concerns about mitochondrial dysfunction in renal insufficiency (18); while VEGFR2 inhibition may reduce pathological angiogenesis, EMA data link baricitinib to increased DVT risk, particularly in vulnerable populations (19, 20); Although inhibiting the JAK/STAT pathway can inhance glutathione levels and reduce oxidative stress, long-term use is associated with reversible declines in hemoglobin (-0.15 g/dL) and neutrophil counts (21, 22). These findings suggest target-specific organ effects requiring mechanistic clarification.

The clarification of such target-specific effects demands research strategies that transcend conventional validation paradigms, particularly for a systemic disease like CKD. For kinase inhibitors like baricitinib, mechanistic understanding has traditionally been built upon discrete *in vitro* assays. Key examples include employing fluorescence resonance energy transfer (FRET)-based biosensors to monitor and quantify its inhibition of IL-6/JAK/STAT signaling dynamics in live cells (23), using flow cytometric phospho-specific analysis to quantify its dose-dependent inhibition of STAT3 phosphorylation (24), and conducting specific kinase activity assays to demonstrate its potent inhibition of JAK2 enzymatic function (25). While these methods are foundational for verifying predefined, singular targets, they offer limited capacity for the unbiased exploration of unanticipated polypharmacology, specifically the simultaneous engagement of multiple targets within the interconnected pathways that characterize CKD.

To address these challenges, we propose an integrated safety framework. This framework combines computational prediction, structural validation, and real-world evidence to enable a comprehensive benefit-risk assessment. Network toxicology is employed to integrate genomic and proteomic data, enabling the construction of a baricitinib-CKD interaction network to identify critical therapeutic and toxicity nodes. Building on these predictions, molecular docking is applied to validate target binding modes and affinity at the atomic level. Future studies could employ molecular dynamics simulations to further elucidate the binding dynamics and stability of these complexes, building on the docking results presented here. Finally, systematic mining of the FAERS database collects baricitinib-related adverse drug events (ADEs) and analyzes reporting odds ratios (RORs) for major AEs. This three-tiered approach systematically bridges predicted molecular interactions, structural docking validation, and epidemiological evidence, creating an integrative analysis from predicted binding modes to population health outcomes. Thus, this framework aims to generate CKD-specific mechanistic hypotheses via target-disease intersection, and to interpret these hypotheses within the context of safety signals observed from the drug’s broader clinical use.

The framework aims to generate and prioritize mechanistic hypotheses regarding baricitinib’s potential multi-target effects on inflammation and fibrosis in CKD. By systematically bridging computational predictions with real-world safety evidence, this integrated approach provides a rational foundation for designing personalized dosing strategies and risk mitigation plans. Thus, it advances the paradigm toward data-driven, precision application of JAK inhibitors in nephrology.

## 2 Materials and methods

### 2.1 Network toxicology analysis

In this study, computational toxicology techniques were employed to predict the toxicity of baricitinib. The compound’s canonical SMILES structure code (CCS( = O)( = O)N1CC(C1)(CC#N) N2C = C(C = N2)C3 = C4C = CNC4 = NC = N3) was retrieved from the PubChem database.^1^ Subsequently, systematic evaluations were carried out using the ProTox 3.0^2^ and ADMETlab 2.0^3^ prediction platforms. These two platforms generate multi—dimensional toxicity parameter data by leveraging the compound’s structural features and established toxicity prediction models. By cross-validating the prediction results of the two platforms, a preliminary cross-validation framework for the toxicological characteristics of baricitinib was constructed.

### 2.2 Collection of baricitinib targets

To comprehensively identify the potential action targets of baricitinib, data from multiple databases were integrated in this study. First, based on the canonical SMILES code obtained, known targets were retrieved from the ChEMBL database^4^ with Homo sapiens specified as the species. Subsequently, the SMILES sequence was simultaneously submitted to the STITCH^5^ and SwissTargetPrediction^6^ platforms for complementary target prediction, aiming to uncover potentially overlooked interacting targets. After cross—validating the prediction results of the three databases, the UniProt database^7^ was utilized to standardize the target names. Then, data from multiple platforms were combined, and non-specific targets were filtered out. Finally, a structurally complete and data—consistent target dataset was established.

### 2.3 Selection of CKD—related target networks

This study integrated resources from three authoritative databases: GeneCards,^8^ OMIM,^9^ and the Therapeutic Target Database (TTD).^10^ These databases contain large-scale multi-omics data on the associations between human genes and genetic diseases, enabling systematic screening of potential key targets in CKD. During data processing, differentiated screening strategies were applied based on database-specific characteristics. For GeneCards, a strict screening threshold of “Relevance score > 10” was imposed using its quantitative evaluation metrics. As OMIM and TTD lack quantitative scoring systems, their raw search results were retained to ensure data integrity. A Venn diagram was then employed to analyze and identify overlapping targets between baricitinib’s action targets and CKD-related targets, with intersecting nodes designated as potential drug targets for CKD intervention. The remaining 5,503 targets (5,732–229) represent CKD-specific pathological factors not directly modulated by baricitinib, while the 166 baricitinib-exclusive targets (395–229) reflect non-renal pharmacological actions; both were excluded from further mechanistic analysis. This approach prioritizes targets with direct drug-disease interaction for network construction, ensuring biological relevance. Following systematic target analysis, an interaction network between baricitinib and CKD was constructed to characterize its multi-target action profile. Additionally, Cytoscape (version 3.10.0) was utilized to visualize a compound-target-disease network based on the curated target information. This network toxicology strategy, which intersects a broad CKD gene set with predicted drug targets, is designed to generate testable hypotheses and prioritize key signaling nodes for further investigation. By highlighting pathways commonly implicated in fibro-inflammatory processes, it provides a systems-level roadmap to guide subsequent mechanistic validation in the specific context of CKD. This network toxicology strategy, therefore, serves as a foundational, hypothesis-generating step to identify candidate mechanisms for subsequent validation.

### 2.4 Construction of protein—protein interaction networks and screening of core targets

To characterize the drug-target interaction network, common action genes of baricitinib and CKD were submitted to the STRING online platform,^11^ with species restricted to Homo sapiens. The STRING database predicts functional associations between proteins, supported by multiple types of evidence, and assigns each a confidence score (0–1). In the resulting network, the nodes, representing proteins, are colored to distinguish query proteins from their interactors, while the connecting edges are color-coded to indicate the source of the supporting evidence (26). A medium confidence threshold (> 0.4) was applied to filter protein-protein interaction (PPI) relationships, and disconnected nodes were removed from the network visualization. Following acquisition of preliminary interaction data, Cytoscape was employed to construct a PPI network diagram, with node connectivity parameters calculated using the software’s built-in algorithms. Additionally, core targets of baricitinib in CKD were identified using a threshold of degree value > twice the median (27).

### 2.5 Enrichment analysis

Functional enrichment analysis was carried out using the clusterProfiler package in R. Target gene identifiers were standardized via the org.Hs.eg.db human gene annotation database. The stringr package was employed for differential gene expression data cleaning, aiming to systematically dissect the molecular mechanisms underlying baricitinib’s effects on CKD. Gene Ontology (GO) analysis encompassed three functional domains: biological process (BP), molecular function (MF), and cellular component (CC). A dual-significance threshold was applied (*p* < 0.05; false discovery rate (FDR)-adjusted *p* < 0.05, based on the Benjamini–Hochberg method), with the top 10 significantly enriched terms retained for each subclass according to adjusted *p*-value ranking. The gene ratio, defined as the proportion of genes enriched in each term relative to the total number of input genes, was also reported for each result. For Kyoto Encyclopedia of Genes and Genomes (KEGG) pathway analysis, the top 30 enriched pathways were selected. Multidimensional visualizations were generated using the ggplot2, ggpubr, and circlize R packages, intuitively illustrating how the drug regulates disease phenotypes through coordinated biological processgulates dzations were generated.

### 2.6 Molecular docking validation

In this study, the top five key targets ranked by degree value were selected for molecular docking validation with baricitinib. The SDF structure file of baricitinib was first retrieved from PubChem. Core target protein structures in Protein Data Bank (PDB) format were then downloaded from the RCSB PDB database^12^ using their UniProt IDs. Docking analyses were performed using the CB-Dock2 platform,^13^ which integrates CurPocket curvature-based cavity detection with AutoDock Vina. The platform automatically performs ligand and target protein hydrogenation, dehydration, energy optimization, and structure minimization, then identifies large surface cavities as potential binding sites to generate multiple docking conformations. CB-Dock2 selects the docking pose with the lowest Vina score, calculates binding energy, and generates 3D visualizations of binding modes to systematically characterize molecular interactions between baricitinib and core target proteins. Molecular docking against the canonical targets JAK1 and JAK2 was conducted under identical parameters as a positive control.

### 2.7 FAERS data acquisition and ADE screening

This study utilized data sourced from the FAERS database, an internationally recognized open drug safety surveillance platform that aggregates spontaneous AE reports from global consumers, healthcare providers, and pharmaceutical entities. Post-market full-cycle monitoring data (2018–2024) were selected based on baricitinib’s FDA approval timeline, encompassing the complete temporal spectrum of drug safety signals. Raw data were downloaded in American Standard Code for Information Interchange (ASCII) report format, comprising ADE reports designating baricitinib as the primary suspect medication. Ultimately, an analytical dataset was constructed containing key variables such as report date, patient age and gender, reporter type, and geographic region. This dataset reflects post-marketing safety experience across baricitinib’s approved indications.

### 2.8 Data preprocessing and standardization

**FIGURE 1:**
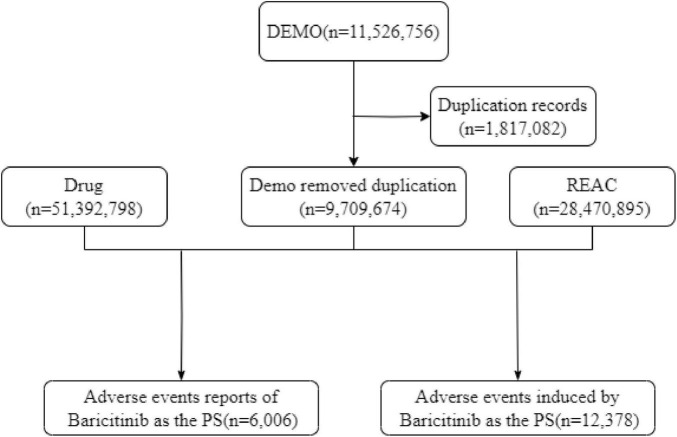
Flowchart illustrating the process for selecting baricitinib–associated AEs from FAERS.

Drug nomenclature was standardized using Medex UIMA 1.3.8 (28), and adverse reaction terminology was classified and coded according to the Medical Dictionary for Regulatory Activities (MedDRA) 26.1 standard (29), which organizes reports into hierarchical categories: Standardized MedDRA Organ Classes (SOCs) representing organ systems, and Preferred Terms (PT) describing specific AEs. A rigorous data cleaning pipeline was implemented for quality control: duplicate reports were identified and removed (retaining the most recent record for identical CASEIDs) (30), outliers were rectified, and field formats were standardized to ensure data consistency and reliability. Following preprocessing, the dataset comprised 9,709,674 demonstration reports, encompassing 51,392,798 drug cases and 28,470,895 REAC records (Figure 1). Statistical analyses were performed using R software (version 4.4.2), and data flow diagrams were visualized via draw.io.

### 2.9 Disproportionality analysis and signal detection

In this study, the RORs from disproportionality analysis were employed to evaluate ADE signals. To ensure result comprehensiveness and accuracy, gender stratification was conducted to separately assess ROR values in male and female populations. The utility of ROR resides in its capacity to correct biases arising from low reporting frequencies of specific events. Compared to ROR, the advantage of the Proportional Reporting Ratio (PRR) is its higher specificity. This study employed both ROR and PRR methods to leverage their respective strengths, aiming to detect more reliable safety signals. ROR and PRR were calculated according to Table 1, with a positive ADE signal defined as meeting all the following criteria: a ≥ 3, ROR 95% CI (lower limit) > 1, and PRR 95% CI (lower limit) > 1. Higher values of ROR and PRR indicate a stronger ADE signal intensity (31).


$$\mathrm{ROR}=\frac{\text{ad}}{\text{bc}}$$

$$95\%\,\mathrm{CI}={\text{e}}^{\ln\left(\text{ROR}\right)\pm 1.96\,\sqrt{\left(\frac{1}{\text{a}}+\frac{1}{\text{b}}+\frac{1}{\text{c}}+\frac{1}{\text{d}}\right)}}$$

$$P\,R\,R=\frac{a/\left(a+b\right)}{c/\left(c+d\right)}$$

$$95\%\,C\,I=e^{\ln\left(\text{PRR}\right)\pm 1.96\,\sqrt{\left(\frac{1}{a}-\frac{1}{a+b}+\frac{1}{c}-\frac{1}{c+d}\right)}}$$

**TABLE 1:** Contingency table for disproportionality analysis.

Drug exposure	ADE present	ADE absent	Total
Baricitinib	a	b	a + b
Non-Baricitinib	c	d	c + d
Total	a + c	b + d	*N* = a + b + c + d

## 3 Results

### 3.1 Computational toxicity prediction for baricitinib

Based on the integrated analysis of ProTox3.0 and ADMETlab2.0, the toxicity risk profile of baricitinib is as follows: Respiratory toxicity and acute toxicity were clearly categorized as high risk. Hepatotoxicity and neurotoxicity showed predictive discrepancies between platforms and were collectively determined to be low risk. Carcinogenicity exhibited consistent low-risk predictions across both platforms. No risk signals were detected for nephrotoxicity or cardiotoxicity. Both platforms yielded negative results for eye corrosion and skin sensitization (Table 2).

**TABLE 2:** Integrated toxicity risk assessment of baricitinib using ProTox3.0 and ADMETlab2.0 computational platforms.

Toxicity endpoint	ProTox3.0 prediction	ADMETlab2.0 prediction	Integrated risk category
Hepatotoxicity	-	+	+
Neurotoxicity	+	–	+
Nephrotoxicity	–	ND	–
Respiratory toxicity	++	+	++
Cardiotoxicity	–	ND	–
Carcinogenicity	+	+	+
Eye corrosion	–	–	–
Skin sensitization	–	–	–
Acute toxicity	–	++	++

The integrated risk category is determined by harmonizing predictions from ProTox3.0 and ADMETlab2.0. Symbols indicate: - (Negative, agreement between platforms or single negative prediction with insufficient supplementary data), **+** (Low-risk, single positive prediction with probability 0.3–0.7 or prioritized high-risk evidence in conflicts), **++** (High-risk, dual positive predictions or single prediction with probability ≥ 0.7). ND, No data available.

### 3.2 Integrated analysis of baricitinib targets and CKD-related genes

**FIGURE 2:**
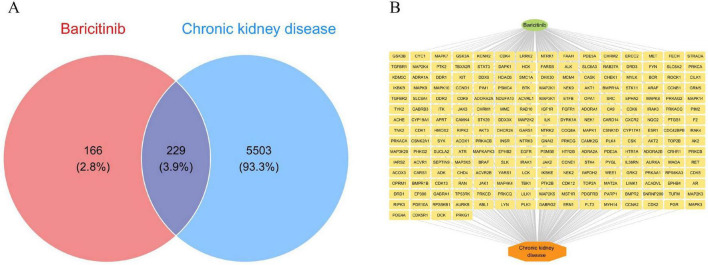
**(A)** Venn diagram illustrating the intersection between baricitinib’s potential targets (395, left circle) and CKD-related genes (5,732, right circle), with 229 shared targets identified through multi-database integration. Non-overlapping areas: 5,503 CKD-exclusive targets (right) and 166 baricitinib-exclusive targets (left), excluded to prioritize direct drug-disease interactions. **(B)** Network graph visualizing the complex regulatory relationships of baricitinib (central node) with its key targets (middle-layer nodes) and CKD pathological processes (outer-layer nodes), providing a global perspective for deciphering baricitinib’s multi-target synergistic mechanisms.

This study systematically examined baricitinib’s potential targets in CKD using a multi−source data integration approach. Initially, drug target databases such as ChEMBL, STITCH, and SwissTargetPrediction were queried to screen 395 molecular targets potentially interacting with baricitinib. Concurrently, 5,732 disease genes closely associated with CKD pathological processes were retrieved from disease databases including GeneCards, OMIM, and TTD. Through data cleansing and cross-comparison analysis, 229 common targets of significant research value were ultimately identified, constituting a key molecular network through which baricitinib might modulate CKD pathological processes. Visual analysis using a Venn diagram intuitively illustrated the intersection between baricitinib targets and CKD-related genes, offering novel research directions for in-depth exploration of its mechanism of action (Figure 2A). Additionally, a compound-target-disease network was depicted in Figure 2B.

### 3.3 Interaction networks of potential targets and identification of core genes

**FIGURE 3:**
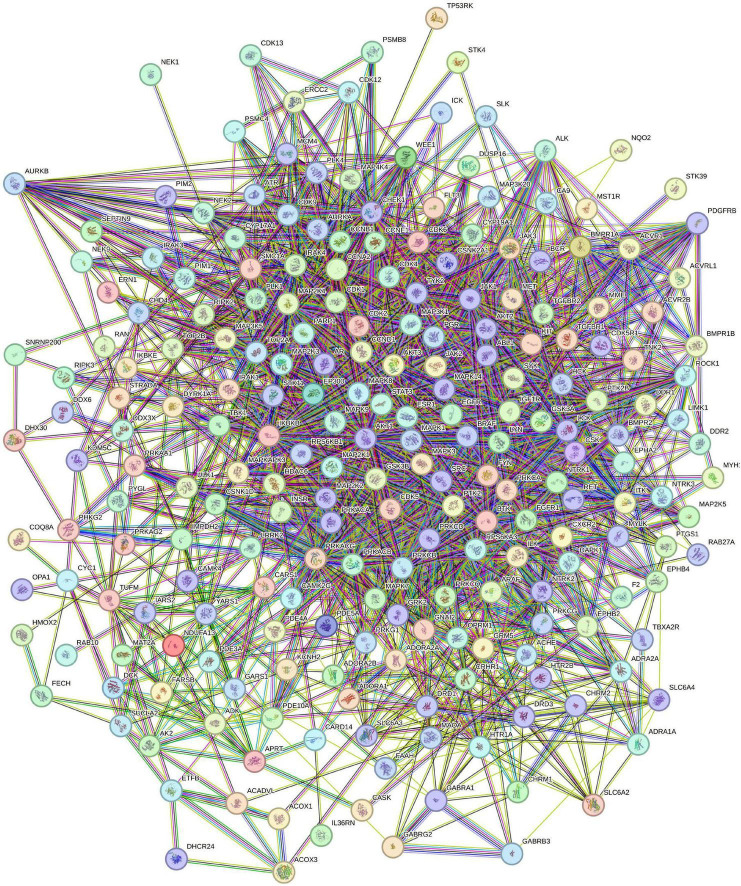
Protein-protein interaction (PPI) network of shared targets between baricitinib and chronic kidney disease. The network was generated using the STRING database (confidence threshold > 0.4) and comprises 229 nodes and 2,350 edges. Network nodes represent proteins. Edges represent predicted protein-protein associations. Edge colors indicate the source of interaction evidence, as defined by STRING: from curated databases (cyan blue); experimentally determined (purple); gene neighborhood (green); gene fusions (red); protein co-occurrence (blue); text mining (yellow); co-expression (black); protein homology (pale blue). Colored nodes represent query proteins and first shell of interactors. Filled nodes have a known or predicted 3D structure.

**FIGURE 4:**
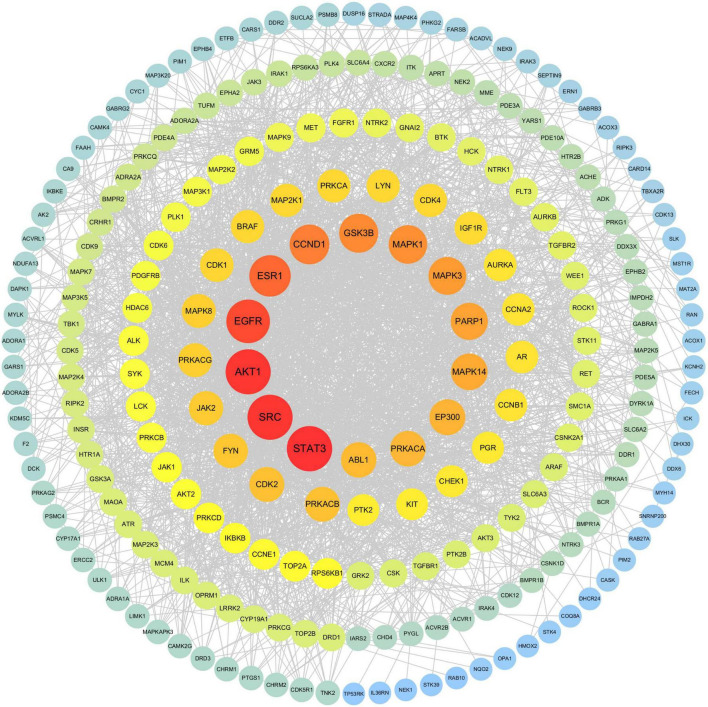
The protein-protein interaction (PPI) network of candidate targets was visually refined in Cytoscape to clearly show functional connections; node size and color intensity represent their relative significance, while the thickness and color intensity of edges mirror the confidence level of interactions. This multi-parameter visualization approach not only vividly showcases the network hubness of core targets but also facilitates the swift identification of high-weight interaction relationships within key regulatory pathways via differences in graph attributes.

**FIGURE 5:**
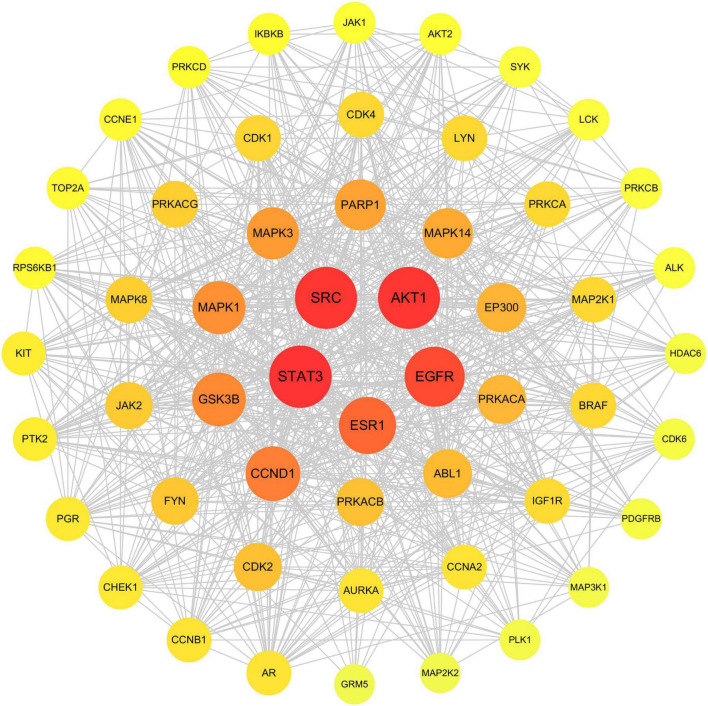
The PPI network of core targets focuses on the intricate interaction patterns among 53 key targets. Topological analysis of the network identified AKT1, EGFR, ESR1, SRC, and STAT3 as nodes with the highest degree values. In the visualization, these targets are highlighted by enlarged node sizes and intensified red saturation.

A PPI network was constructed using the STRING database (Figure 3), consisting of 229 nodes and 2,350 interaction edges with an average node degree of 20.5. The TSV-format data were then imported into Cytoscape for visual optimization, yielding a refined PPI network diagram (Figure 4). Leveraging built-in topological analysis algorithms with a threshold of degree value > twice the median, 53 core targets associated with baricitinib’s regulation of CKD were identified. A dedicated PPI network (Figure 5) was generated to visualize interactions among these core targets. Notably, the five most critical targets ranked by degree values were AKT1, EGFR, ESR1, SRC, and STAT3, underscoring their hub roles in network information transmission. Topological properties were computed using the software’s Analyze Network function (Table 3).

**TABLE 3:** Topological features of the five primary hub targets identified from the PPI network.

Gene	Degree	Closeness centrality	Betweenness centrality	Topological coefficient
STAT3	95	0.608579	0.077739	0.156165
AKT1	94	0.610215	0.077326	0.149283
SRC	94	0.597368	0.071724	0.153326
EGFR	88	0.589610	0.058228	0.162806
ESR1	79	0.576142	0.044246	0.174280

### 3.4 Pathway enrichment analysis of core targets

**FIGURE 6:**
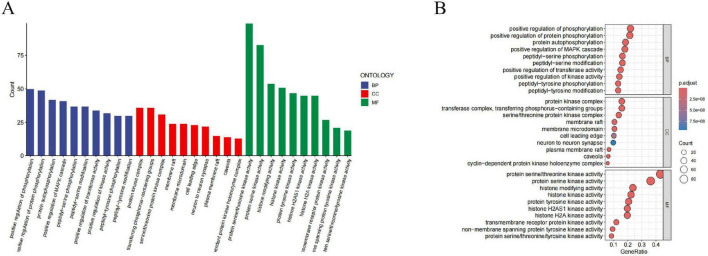
GO enrichment analysis of baricitinib–CKD shared targets (top 10 terms). **(A)** Bar plot displaying the top 10 significantly enriched GO terms across Biological Process (BP), Cellular Component (CC), and Molecular Function (MF) domains, based on FDR-adjusted *p* < 0.05. Bar height represents gene counts, while darker color intensity reflects greater statistical significance. **(B)** Bubble plot illustrating enriched GO terms: bubble size denotes the number of enriched genes, and color intensity corresponds to -log_10_(adjusted *p*-value). Key functional categories highlight biological mechanisms potentially modulated by baricitinib in CKD.

GO enrichment analysis revealed that baricitinib’s intervention in CKD is achieved through multidimensional regulation of BP, CC and MF (Figure 6). At the BP level, significantly enriched pathways included positive regulation of phosphorylation (adjusted *p* = 1.48 × 10^–29^; gene ratio = 0.22), positive regulation of MAPK cascade (adjusted *p* = 8.33 × 10^–21^; gene ratio = 0.18), positive regulation of kinase activity (adjusted *p* = 1.55 × 10^–20^, gene ratio = 0.14) and peptidyl-serine phosphorylation (adjusted *p* = 6.80 × 10^–34^; gene ratio = 0.16). CC analysis demonstrated significant aggregation of membrane microdomain (adjusted *p* = 4.66 × 10^–12^; gene ratio = 0.10) and membrane raft (adjusted *p* = 4.66 × 10^–12^; gene ratio = 0.10), along with enrichment of neuron to neuron synapse (adjusted *p* = 9.42 × 10^–8^; gene ratio = 0.10). At the MF level, significant enrichment was observed for protein serine/threonine kinase activity (adjusted *p* = 7.33 × 10^–100^; gene ratio = 0.43), transmembrane receptor protein kinase activity (adjusted *p* = 1.84 × 10^–30^; gene ratio = 0.12) and non-membrane spanning protein tyrosine kinase activity (adjusted *p* = 4.60 × 10^–27^; gene ratio = 0.09).

**FIGURE 7:**
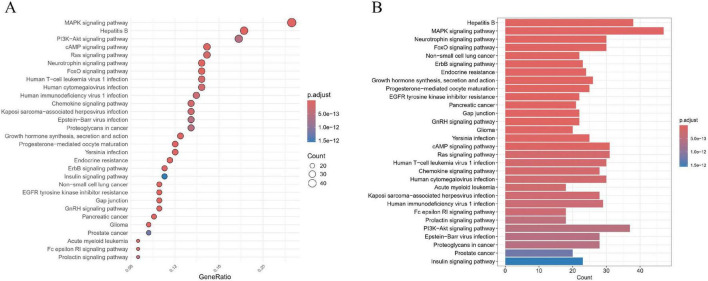
KEGG enrichment analysis of baricitinib-CKD shared targets (top 30 pathways). **(A)** Bubble plot showing enriched KEGG pathways with FDR-adjusted *p* < 0.05. Bubble size indicates gene counts, and color gradient (from light to dark) represents increasing statistical significance (-log_10_ adjusted *p*-value). **(B)** Corresponding bar plot summarizing pathway enrichment results. Bar length reflects gene counts, while color shading denotes adjusted *p*-value magnitude. Highlighted pathways are closely associated with baricitinib’s regulatory effects in CKD.

KEGG enrichment analysis elucidated the key molecular mechanisms underlying baricitinibme intervention in CKD (Figure 7). Significantly enriched pathways included the MAPK signaling pathway (adjusted *p* = 1.42 × 10^–23^; gene ratio = 0.23), PI3K-Akt signaling pathway (adjusted *p* = 5.22 × 10^–13^; gene ratio = 0.18), Ras signaling pathway (adjusted *p* = 1.53 × 10^–13^; gene ratio = 0.15), FoxO signaling pathway (adjusted *p* = 2.01 × 10^–19^; gene ratio = 0.14), Chemokine signaling pathway (adjusted *p* = 1.88 × 10^–13^; gene ratio = 0.13), Human immunodeficiency virus 1 infection (adjusted *p* = 2.68 × 10^–13^; gene ratio = 0.14) and Epstein–Barr virus infection (adjusted *p* = 5.37 × 10^–13^; gene ratio = 0.13).

In the pathway screening process, the above results were grounded not only in statistical significance (complete data for all pathways are provided in Supplementary material) but also prioritized biological pathways directly associated with core CKD pathological mechanisms (e.g., inflammation, fibrosis, and metabolic imbalance). Key targets with therapeutic intervention potential were screened across multi-omics dimensions to ensure alignment between the statistical robustness and clinical translational value of the analysis, thereby providing a verifiable data foundation for subsequent mechanistic investigations.

### 3.5 Molecular docking

**FIGURE 8:**
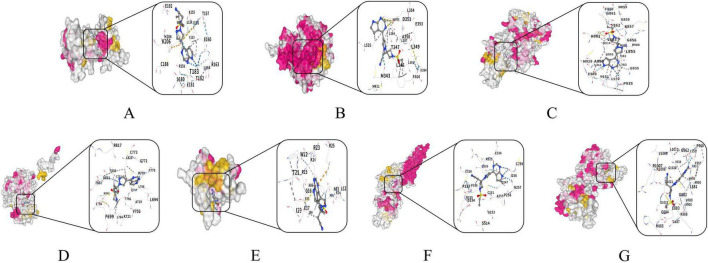
Molecular docking results illustrating the lowest Vina score for each target protein in complex with baricitinib. **(A)** Baricitinib and SRC, **(B)** baricitinib and ESR1, **(C)** baricitinib and JAK2, **(D)** baricitinib and EGFR, **(E)** baricitinib and AKT1, **(F)** baricitinib and STAT3, **(G)** baricitinib and JAK1.

Table 4 compiles detailed molecular docking results between baricitinib and five core targets: AKT1, EGFR, ESR1, SRC and STAT3, alongside its canonical targets JAK1 and JAK2 included as a positive control. Automated analysis via the CB-Dock2 platform revealed binding free energies ranging from –6.9 to –8.4 kcal/mol for these hubs. Binding to JAK1 and JAK2 was also strong (–6.3 and –7.6 kcal/mol, respectively), validating the protocol. Notably, the docking scores suggested comparable or stronger theoretical binding of baricitinib to these predicted hubs than to its canonical target JAK1. A binding energy below 0 kcal/mol indicates molecular binding activity, with lower values reflecting stronger interactions. In this study, all energy values far exceeded the strong binding threshold of –5.0 kcal/mol, fully demonstrating highly spontaneous and stable interactions between baricitinib and these targets. Figure 8 depicts the lowest-energy conformation in three dimensions, highlighting key amino acid residues participating in hydrogen bond formation.

**TABLE 4:** The molecular docking results of key targets and baricitinib.

Compound	Target	Vina Score (kcal/mol)
Baricitinib	SRC	–8.4
Baricitinib	ESR1	–8.2
Baricitinib	JAK2	–7.6
Baricitinib	EGFR	–7.5
Baricitinib	AKT1	–7.0
Baricitinib	STAT3	–6.9
Baricitinib	JAK1	–6.3

### 3.6 Basic characteristics of baricitinib-related ADEs in the FAERS database

Based on data from the US FAERS (2018–2024), a total of 6,006 ADEs reports related to baricitinib were screened. Among reports with recorded gender information, males constituted a higher proportion (62.65%) compared to females (28.84%), while 511 reports (8.51%) had no gender information reported. Regarding age distribution, the proportion of reports from patients under 45 years old was relatively low ( < 18 years: 1.18%; 18–45 years: 11.49%), whereas the proportion of those over 45 years old increased significantly (45–65 years: 28.27%; ≥ 65 years: 29.37%), although 29.69% of reports lacked age data. Reporter types were predominantly Consumers (53.63%), followed by Pharmacists (22.28%) and Physicians (13.64%). Reports originated from multiple countries across different regions, with the United States accounting for the highest proportion (55.73%), followed by Japan (10.29%), the United Kingdom (4.43%), France (4.30%), and Germany (4.06%), while reports from other countries were fewer. In terms of clinical outcomes, the most common result was “Other serious” (40.74%), followed by Hospitalization (36.08%) and Death (11.50%). Detailed information is presented in Table 5.

**TABLE 5:** Basic information on baricitinib−related ADEs was extracted from the FAERS database.

Factors	Total events (%)
**Year**	
2018	207 (3.45)
2019	851 (14.17)
2020	660 (10.99)
2021	1,221 (20.33)
2022	931 (15.50)
2023	1,150 (19.15)
2024	986 (16.42)
**Gender**	
Female	3,763 (62.65)
Male	1,732 (28.84)
Unknown	511 (8.51)
**Age (year)**	
< 18	71 (1.18)
18∼45	690 (11.49)
45∼65	1,698 (28.27)
≥ 65	1,764 (29.37)
Unknow	1,783 (29.69)
Weight (kg)	80.00 (64.70, 97.52)
**Reporter**	
Consumer	3,221 (53.63)
Pharmacist	1,338 (22.28)
Physician	819 (13.64)
Lawyer	1 (0.02)
Other health-professional	161 (2.68)
Unknown	466 (7.76)
**Reported countries**	
United States	3,347 (55.73)
Japan	618 (10.29)
United Kingdom	266 (4.43)
France	258 (4.30)
Germany	244 (4.06)
Italy	162 (2.70)
Spain	109 (1.81)
China	76 (1.27)
Sweden	73 (1.22)
Brazil	70 (1.17)
Austria	53 (0.88)
Australia	50 (0.83)
Other	680 (11.32)
**Route**	
Oral	3,764 (62.64)
Subcutaneous	10 (0.17)
Other	2,235 (37.19)
**Outcomes**	
Hospitalization	1,644 (36.08)
Death	524 (11.50)
Life threatening	339 (7.44)
Disability	95 (2.09)
Required intervention to prevent permanent impairment/damage	94 (2.06)
Congenital anomaly	4 (0.09)
Other serious	1,856 (40.74)
**AE occurrence time (days)**	
< 7	530 (12.98)
7∼28	423 (10.36)
28∼60	144 (3.53)
≥ 60	728 (17.83)
Unknow	2,259 (55.31)

### 3.7 Baricitinib signal mining

Through analysis of baricitinib AE reports from the general FAERS population, this study identified 24 SOCs. Results indicated that infections and infestations [*n* = 2,227, ROR = 3.57 (95% CI: 3.41–3.74), PRR = 3.11 (95% CI: 2.99–3.23)], vascular and lymphatic diseases [*n* = 558, ROR = 2.38 (95% CI: 2.18–2.59), PRR = 2.31 (95% CI: 2.14–2.50)] and respiratory system diseases [*n* = 1,005, ROR = 1.77 (95% CI: 1.66–1.89), PRR = 1.71 (95% CI: 1.61–1.81)] exhibited the highest reporting frequencies and signal intensities (Table 6). Notably, disproportionality analysis did not generate a positive safety signal for renal and urinary disorders [*n* = 225, ROR = 0.86 (95% CI: 0.75–0.98), PRR = 0.86 (95% CI: 0.75–0.99)].

**TABLE 6:** The signal intensities of ADEs of baricitinib at the SOC level in FAERS database.

SOC	Case reports	ROR (95% CI)	PRR (95% CI)
Infections and infestations	2,227	3.57 (3.41, 3.74)	3.11 (2.99, 3.23)
Vascular disorders	558	2.38 (2.18, 2.59)	2.31 (2.14, 2.5)
Respiratory, thoracic and mediastinal disorders	1,005	1.77 (1.66, 1.89)	1.71 (1.61, 1.81)
Investigations	1,192	1.66 (1.57, 1.77)	1.6 (1.51, 1.7)
Cardiac disorders	407	1.6 (1.45, 1.76)	1.58 (1.43, 1.74)
Blood and lymphatic system disorders	321	1.47 (1.32, 1.64)	1.46 (1.3, 1.64)
Hepatobiliary disorders	159	1.46 (1.25, 1.71)	1.45 (1.24, 1.7)
Neoplasms benign, malignant and unspecified (incl cysts and polyps)	592	1.39 (1.28, 1.51)	1.37 (1.27, 1.48)
Musculoskeletal and connective tissue disorders	573	0.87 (0.8, 0.95)	0.88 (0.81, 0.95)
Renal and urinary disorders	225	0.86 (0.75, 0.98)	0.86 (0.75, 0.99)
Gastrointestinal disorders	870	0.83 (0.78, 0.89)	0.84 (0.79, 0.89)
Ear and labyrinth disorders	44	0.82 (0.61, 1.11)	0.83 (0.62, 1.11)
Nervous system disorders	780	0.8 (0.74, 0.86)	0.81 (0.76, 0.86)
Skin and subcutaneous tissue disorders	584	0.75 (0.69, 0.82)	0.76 (0.7, 0.82)
General disorders and administration site conditions	1,469	0.6 (0.57, 0.64)	0.65 (0.63, 0.68)
Metabolism and nutrition disorders	148	0.58 (0.49, 0.68)	0.58 (0.5, 0.68)
Eye disorders	139	0.55 (0.47, 0.65)	0.56 (0.48, 0.66)
Immune system disorders	84	0.52 (0.42, 0.65)	0.53 (0.43, 0.66)
Reproductive system and breast disorders	42	0.52 (0.38, 0.7)	0.52 (0.39, 0.7)
Injury, poisoning and procedural complications	734	0.44 (0.41, 0.48)	0.48 (0.44, 0.52)
Endocrine disorders	12	0.35 (0.2, 0.61)	0.35 (0.2, 0.62)
Congenital, familial and genetic disorders	11	0.32 (0.18, 0.58)	0.32 (0.18, 0.58)
Psychiatric disorders	191	0.27 (0.24, 0.31)	0.28 (0.24, 0.32)
Pregnancy, puerperium and perinatal conditions	11	0.23 (0.13, 0.42)	0.23 (0.13, 0.41)

At the PT level, disproportionality analysis identified multiple high-strength signals. For enhanced clarity, these signals are grouped into three major clinical themes: infections, thrombotic events, and malignancies, with selected key signals detailed below and in Table 7. In the infections category, the strongest signals were sputum culture positive [*n* = 21, ROR = 234.77 (95% CI: 149.80–367.95), PRR = 234.38 (95% CI: 149.33–367.88)], stenotrophomonas test positive [*n* = 4, ROR = 204.43 (95% CI: 73.51–568.51), PRR = 204.37 (95% CI: 73.75–566.30)], and herpes zoster meningitis [*n* = 7, ROR = 153.36 (95% CI: 71.34–329.67), PRR = 153.27 (95% CI: 71.36–329.18)]. For thrombotic events, substantial signals included cerebral artery thrombosis [*n* = 4, ROR = 45.10 (95% CI: 16.76–121.33), PRR = 45.08 (95% CI: 16.92–120.11)], pulmonary artery thrombosis [*n* = 5, ROR = 37.22 (95% CI: 15.38–90.07), PRR = 37.20 (95% CI: 15.40–89.87)], and pulmonary infarction [*n* = 10, ROR = 41.99 (95% CI: 22.46–78.50), PRR = 41.95 (95% CI: 22.40–78.55)]. Among malignancies, notable signals were diffuse large B-cell lymphoma stage IV [*n* = 4, ROR = 55.09 (95% CI: 20.43–148.52), PRR = 55.07 (95% CI: 20.27–149.64)], bronchial carcinoma [*n* = 4, ROR = 33.57 (95% CI: 12.51–90.11), PRR = 33.56 (95% CI: 12.60–89.42)], and adenocarcinoma gastric [*n* = 7, ROR = 32.53 (95% CI: 15.42–68.61), PRR = 32.51 (95% CI: 15.44–68.47)]. The complete dataset encompassing all top 30 signals is available as Supplementary Table 1.

**TABLE 7:** High-strength AEs of baricitinib grouped by clinical theme.

Clinical theme	PTs	Case reports	ROR (95% CI)	PRR (95% CI)
Infections	Sputum culture positive	21	234.77 (149.8, 367.95)	234.38 (149.33, 367.88)
	Stenotrophomonas test positive	4	204.43 (73.51, 568.51)	204.37 (73.75, 566.3)
	Herpes zoster meningitis	7	153.36 (71.34, 329.67)	153.27 (71.36, 329.18)
Thrombotic events	Cerebral artery thrombosis	4	45.1 (16.76, 121.33)	45.08 (16.92, 120.11)
	Pulmonary infarction	10	41.99 (22.46, 78.5)	41.95 (22.4, 78.55)
	Pulmonary artery thrombosis	5	37.22 (15.38, 90.07)	37.2 (15.4, 89.87)
Malignancies	Diffuse large b-cell lymphoma stage iv	4	55.09 (20.43, 148.52)	55.07 (20.27, 149.64)
	Bronchial carcinoma	4	33.57 (12.51, 90.11)	33.56 (12.6, 89.42)
	Adenocarcinoma gastric	7	32.53 (15.42, 68.61)	32.51 (15.44, 68.47)

## 4 Discussion

This exploratory study integrates network toxicology, molecular docking, and pharmacovigilance to develop a multi-dimensional, hypothetical profile of baricitinib in CKD. The discussion that follows aims to synthesize these data into mechanistic hypotheses and to interpret them cautiously within the context of the drug’s established clinical risk profile. Our computational approach identified 229 shared targets between baricitinib and CKD. Molecular docking supported the plausibility of interaction with several core targets (SRC: -8.4 kcal/mol; ESR1: -8.1 kcal/mol; EGFR: -7.5 kcal/mol; AKT1: -7.0 kcal/mol; STAT3: -6.9 kcal/mol). Essentially, the docking scores indicated more favorable predicted binding for these hubs than for JAK1 (-6.3 kcal/mol). The significant topological centrality of hub proteins AKT1 (degree = 94), SRC (94), and STAT3 (95) underscores their critical involvement in CKD pathogenesis. This predicted multi-target engagement profile may underlie the improvements in renal fibrosis and albuminuria observed when baricitinib was administered in preclinical models of CKD, including 5/6 Nx and unilateral ureteral obstruction (12–14). Our analysis thus extends these phenotypic observations by proposing a broader polypharmacology mechanism that encompasses key regulators of inflammation and fibrosis beyond the canonical JAK-STAT axis. AKT1 and EGFR collaboratively activate PI3K-Akt-mediated fibrogenic pathways, inducing collagen IV overexpression that promotes extracellular matrix (ECM) deposition. Concurrently, SRC and STAT3 orchestrate JAK-STAT-dependent inflammatory cascades characterized by dysregulated IL-6 and TNF-α secretion. Complementing these mechanisms, ESR1 fine-tunes Nrf2-modulated oxidative stress responses. This multi-target network establishes an integrated pathological axis driving renal interstitial fibrosis progression and tubular atrophy development (32–36).

Functional enrichment analyses highlight potential pathways and biological processes that could be relevant to baricitinib’s putative effects in CKD. Our integrated computational approach consistently identified the JAK-STAT and MAPK signaling cascades as central targets. The high-affinity molecular docking of baricitinib with core regulators within these pathways, notably STAT3 and SRC, provides structural validation for this predicted engagement. These findings mechanistically explain how baricitinib attenuates ERK/JNK phosphorylation downstream of the JAK-STAT/MAPK signaling axis, thereby reducing pro-inflammatory cytokine release from renal tubules and ameliorating tubulointerstitial inflammatory injury (37). The enrichment of phosphorylation-related biological processes aligns with the potential to disrupt pro-fibrotic signaling, such as the TGF-β1/Smad3 axis (38). CC findings implicate specialized membrane microdomains in the modulation of inflammatory signal transduction (39–41). Furthermore, MF analysis supports a dual mechanism of action, concurrently targeting pathways involved in ECM synthesis and those driving EMT (42, 43). Also, the enrichment of non-membrane spanning protein tyrosine kinase activity requires cautious interpretation, as this GO category encompasses membrane-anchored kinases (e.g., Src family members), reflecting their broad involvement in intracellular signaling rather than specific targeting of non-transmembrane kinases (44). KEGG pathway analysis corroborates this integrated mechanism, highlighting potential suppression of fibrosis-driving pathways including the MAPK/ERK and PI3K-Akt pathways, alongside the modulation of complementary protective pathways such as the antioxidant FoxO and chemokine signaling pathways (45–48). The enrichment of virus-related pathways further aligns with the drug’s recognized immunosuppressive profile (49). This comprehensive pathway network validates the central role of the MAPK/PI3K-Akt axis while revealing novel Ras/FoxO regulatory mechanisms, providing cross-omics support for multi-target therapeutic strategies. This mechanistic foundation directly informs our risk-stratified clinical approach.

Notably, the high binding stability of SRC and ESR1 suggests their pivotal role in regulating inflammatory microenvironment and oxidative stress (50, 51). Furthermore, pharmacovigilance analysis based on the FAERS revealed dose-dependent risk profiles, including serious infections, thromboembolic events, and tumorigenic risks. Integrated computational toxicology analysis predicted a high-risk profile for respiratory and acute toxicity associated with baricitinib. Hepatotoxicity and neurotoxicity demonstrated predictive divergence between platforms and were conservatively classified as low-risk endpoints. Carcinogenicity exhibited consistent low-risk predictions across both platforms. Nephrotoxicity and cardiotoxicity risk signals were absent. Both platforms yielded negative predictions for eye corrosion and skin sensitization. This discrepancy in acute toxicity predictions highlights the need to prioritize high-confidence signals in integrated risk assessments. Methodologically, this study innovatively established a “computational prediction-molecular validation-real-world evidence” tripartite framework, integrating network toxicology for target screening, molecular docking for binding validation, and FAERS for clinical risk quantification, thereby achieving a comprehensive chain of evidence from molecular mechanisms to population-level effects, overcoming the limitations of traditional single-dimensional research (52, 53). A key aspect of this integrated strategy is the cautious, interpretative synthesis of distinct evidence types: the network toxicology and docking analyses are explicitly tailored to CKD pathophysiology, whereas the FAERS data reflect the drug’s safety profile in its broader clinical use. Clinical inferences for CKD are therefore derived from the convergence of these complementary perspectives.

Based on our computational predictions, we propose a testable hypothesis that baricitinib may attenuate inflammatory and fibrotic signaling in CKD via a central “SRC–EGFR–AKT1” axis. This model, derived from network toxicology and docking affinity data, provides a mechanistic postulate that requires direct experimental validation. Notably, SRC, the highest-affinity target, significantly reduces IL-6/TNF-α secretion by inhibiting the NF-κB/MAPK pathway, thereby remodeling the renal interstitial inflammatory microenvironment (54, 55). Baricitinib selectively inhibits JAK1/2 kinase activity, blocking STAT3 phosphorylation downstream of IL-6 signaling, which reduces pro-inflammatory cytokine production and monocyte migration, ultimately ameliorating renal tubular pathological damage. Intriguingly, persistent STAT3 activation may upregulate negative feedback regulators such as SOCS3, indirectly attenuating JAK-STAT signaling and establishing a bidirectional dynamic regulatory mechanism (56, 57). Concurrently, EGFR interferes with TGF-β1/Smad2/3 signaling, downregulating α-SMA expression to suppress EMT progression (58), while AKT1 inhibits PI3K-Akt-mTOR pathway activity, reducing collagen IV and fibronectin deposition (59). The high affinity of ESR1 suggests its potential role in enhancing antioxidant capacity via Nrf2 pathway activation, though the precise mechanism requires further experimental validation (60). Viewed through the lens of our integrated analysis, the predicted multi-target profile allows us to generate plausible mechanistic hypotheses for the recognized clinical risks associated with baricitinib. Specifically, inhibition of the JAK-STAT pathway could underlie the observed high infection rates by impairing immune surveillance (61); broad modulation of EGFR signaling, potentially via aberrant MAPK pathway activation, might contribute to reported tumor risks (62); and vascular endothelial dysfunction emerges as a candidate mechanism for thromboembolic events (63). Our integrated analysis offers a perspective that extends the conventional view of JAK inhibitors. It proposes a model in which baricitinib could contribute to dual suppression of inflammation and fibrosis in CKD through the predicted multi-target axis described above (64). We further propose a “target affinity-pathway inhibition-clinical risk” model, elucidating the nonlinear relationship between SRC binding energy and infection risk, thereby providing a theoretical basis for dose optimization.

FAERS data analysis provides critical evidence regarding baricitinib’s clinical safety profile, documenting dose-dependent risks of infections and thrombotic events. Especially, no positive disproportionality signal was identified for renal AEs in this dataset. High-risk signals include herpes zoster meningitis and pulmonary artery thrombosis. Disproportionality analysis did not detect a positive safety signal for renal disorders [ROR = 0.86 (95% CI: 0.75–0.98)]. While this is consistent with the drug’s predicted anti-fibrotic profile, it is critical to emphasize that this pharmacovigilance observation does not constitute evidence of nephroprotection. Spontaneous reporting data cannot establish causality, and any potential renoprotective effect remains a hypothesis to be tested in prospective clinical trials in CKD. Nevertheless, by integrating our multi-target mechanistic insights with a clinical safety profile that balances recognized risks of infection and thrombosis against the absence of a disproportionate renal AE signal, we propose a hypothesis-driven, stratified management strategy for future investigation in CKD patients: In patients with advanced CKD, the interplay between reduced renal clearance and predicted high target affinity forms a pharmacokinetic-pharmacodynamic rationale for investigating dose adjustment (65). Future trials in this population could explore personalized dosing strategies as a research question and should be designed to explore strategies that account for individual variability, which may include investigating personalized dose reductions and combination regimens with reno-protective agents (e.g., SGLT-2 inhibitors) to assess the potential for optimizing the benefit-risk ratio (66); Future trials involving CKD patients with comorbidities such as diabetes or hypertension should incorporate rigorous monitoring protocols. These protocols could include pretreatment assessment of herpes virus immunity and coagulation status, with dynamic tracking of biomarkers like D-dimer for thrombosis and indicators of opportunistic infection (67, 68); Furthermore, EGFR-related oncological risks warrant enhanced malignancy screening in long-term users (68). This strategy integrates target affinity and clinical risk intensity, thereby outlining a conceptual framework of dynamic dose–target inhibition–risk thresholds to guide future research on personalized management in CKD and the precision application of JAK inhibitors.

The translational significance of this study lies both in informing personalized management strategies for CKD and in elucidating the molecular rationale that could support the therapeutic repurposing of baricitinib for CKD and other fibrotic conditions. In RA therapy, the drug primarily relies on JAK-STAT pathway inhibition to suppress inflammatory cascades. Conversely, its efficacy in CKD involves a more complex regulatory network—the high binding energy characteristic of SRC kinase results in significantly prolonged tissue half-life (69). Consistent with prior pharmacokinetic modeling (70, 71), dose adjustment may be warranted in patients with advanced CKD to balance target inhibition and minimize risks such as infections. This dose optimization model is directly based on our quantitative analysis of SRC target binding energy. Furthermore, the established dynamic monitoring system utilizes mechanism-guided biomarkers: serum IL-6 receptor saturation (critical value > 85%) can provide early warning of immunodeficiency states induced by JAK-STAT over-inhibition (72), while plasma D-dimer levels quantify SRC-mediated thrombotic risk associated with vascular endothelial dysfunction (73). Together, these biomarkers form a decision-making loop from molecular mechanisms to clinical practice. The biological basis for cross-indication expansion lies in the “microenvironment-dependent regulation” of target functions. Specifically, in RA, SRC promotes invasive migration of synovial fibroblasts through the RhoA/ROCK pathway (74); In CKD, SRC reduces renal tubulointerstitial inflammatory infiltration by negatively regulating the NF-κB/MAPK signaling axis and inhibits collagen deposition via the TGF-βR/EGFR signaling axis (54, 55, 69); while EGFR drives VEGF-mediated pannus formation in RA (75) yet activates TGF-β1/Smad3 phosphorylation to induce EMT in CKD (76). This inferred context-dependence of target function provides a hypothetical molecular framework for investigating its potential application across fibrotic diseases: AKT1 inhibition blocks PI3K-mTOR to reduce hepatic collagen synthesis (77), and EGFR targeting suppresses PDGFR-β autophosphorylation to inhibit alveolar EMT(78), synergizing with CKD’s ECM remodeling. Notably, in systemic sclerosis (SSc), TGF-β drives fibroblast transition via Smad signaling (79), whereas baricitinib’s EGFR affinity downregulates COL1A1/COL3A1 to restore collagen density in skin models (80). Similarly, in non-alcoholic steatohepatitis (NASH), Kupffer cell activation via TLR4 and NLRP3 inflammasomes promotes release of cytokines such as IL-1β and IL-18. This subsequently activates the JAK-STAT pathway, promoting collagen synthesis and fibrosis by hepatic stellate cells (HSCs) (81). Clinical trials have demonstrated that baricitinib treatment significantly reduces hepatic inflammation and fibrosis biomarkers, including P3NP, HA, and TIMP-1 (82). The observed gender-based efficacy differences further underscore the complexity of target regulation. Females exhibit significantly higher PGC-1α activity than males, attributed to elevated ESR1 expression. This heightened activity may confer protection against eGFR decline through enhanced reactive oxygen species (ROS) clearance (83). Conversely, in osteoarthritis (OA) patients, baricitinib suppresses NF-κB phosphorylation, leading to reduced p-p65 levels, attenuated inflammation, and delayed joint destruction(84). This context-dependent specificity is also prominent in neurological disorders. In animal models of experimental autoimmune encephalomyelitis (EAE), baricitinib was confirmed to act as a JAK1/2 inhibitor, delays disease onset, mitigates symptom severity, and inhibits Th1/Th17 polarization alongside STAT1/3/4 phosphorylation. Its mechanism involves diminished release of inflammatory cytokines, alleviating demyelination and immune cell infiltration (85). Within the realm of cardiovascular fibrosis, SRC kinase promotes cardiac fibroblast activation through both Smad2/3-dependent canonical TGF-β1 signaling and MAPK-dependent non-canonical pathways (86). Notably, a 2023 study indicated that baricitinib reduces myocardial collagen volume fraction by 29% (*p* < 0.05) via suppression of these associated pathways (11). In metabolic kidney diseases like DKD, baricitinib exhibits multi-dimensional therapeutic benefits through a triad of mechanisms: anti-inflammatory action, insulin resistance amelioration, and direct antifibrotic effects. Compared with RAAS inhibitor groups, treatment cohorts showed a 21% greater reduction in urinary albumin-to-creatinine ratio (UACR), along with lower 24-h urinary protein excretion and decreased inflammatory markers (87). This cumulative evidence not only validates the scientific rationale for baricitinib’s cross-disease therapeutic potential but also advances the conceptual framework for JAK inhibitors from “single-pathway blockade” toward “multi-dimensional pathological remodeling.”

By combining *in silico* network toxicology predictions, molecular docking validation and real-world pharmacovigilance data, this study systematically analyzed the multi-target synergistic mechanisms of baricitinib in CKD. In contrast to traditional JAK inhibitor studies focusing on single anti-inflammatory pathways, this research unveiled the drug’s dual action mode of synergistically regulating inflammation and fibrosis, offering mechanistic support for expanding the applications of JAK inhibitors in kidney diseases. Methodologically, the dynamic risk model constructed from FAERS data performs correlation analysis between target action characteristics and dose-dependent clinical events (e.g., infections, thrombosis) and integrates multi-omics data to interpret the complex associations between the drugrget action characterfibro genic process and potential risk signals. This provides a quantitative basis for dose optimization and risk monitoring in personalized treatment.

Despite integrating multi-dimensional data, this study has several limitations: First, the pharmacovigilance data originate from baricitinib’s general use, whereas the mechanistic insights are derived from intersecting its predicted targets with a generic CKD gene list—a distinction that necessitates cautious interpretation for direct CKD application. It is imperative to acknowledge the methodological constraints inherent to the FAERS database, which shape the interpretation of all derived safety signals. A fundamental constraint is the lack of reliable exposure denominators (88). Consequently, the observation of no disproportionate signal for renal AEs (ROR < 1), while reassuring, cannot be interpreted as evidence of nephroprotection. This limitation stems from fundamental characteristics of the FAERS database: it does not provide denominators or incidence rates, and reporting patterns are heavily influenced by indication, disease severity, with notable confounding likely arising from the inclusion of patient populations at high intrinsic risk for reported events, such as critically ill individuals with COVID-19, concomitant medications, and spontaneous reporting biases (89, 90). Moreover, the frequent absence of key clinical covariates, including age in a substantial proportion of reports, limits opportunities for adjusted analyses. Our disproportionality screening across numerous Preferred Terms was exploratory and did not include statistical adjustment for multiple testing, a factor that should be considered when evaluating the strength of the associations identified (91). Therefore, an ROR below 1 indicates a relative reporting frequency and is not equivalent to demonstrating a renal protective effect in a CKD population. Furthermore, FAERS relies on voluntary reporting, which may introduce bias in infection and thrombotic event signals, such as the overrepresentation of severe events, and lacks systematic dose-effect data, thereby affecting the accuracy of risk quantification (92), with the voluntary nature of reporting also making these data susceptible to underreporting of mild events and overreporting of severe cases, meaning the signals should be interpreted with caution; Taken together, these constraints underscore that the identified disproportionality signals represent hypothesis-generating safety profiles for focused monitoring, not definitive evidence of causation or incidence. Their clinical interpretation requires integration with evidence from prospective studies, such as controlled trials and observational cohorts with defined populations (93, 94). A fundamental limitation is the use of a broad, systemic CKD gene set, which may not fully capture kidney-specific pathological processes. Thus, the identified pathways indicate potential, non-exclusive points of engagement with CKD-relevant biology (95). This means the enriched pathways, such as the MAPK and PI3K-Akt signaling cascades, reflect baricitinib’s potential engagement with biological processes central to, but not exclusive to, CKD pathogenesis. Thus, the proposed mechanistic model should be viewed as a high-level, integrative hypothesis requiring refinement with kidney-specific data. Second, our target screening was confined to protein-coding genes, excluding non-coding RNAs and epigenetic regulators, which may omit relevant mechanisms (96, 97); Additionally, our molecular docking validation employed static protein structures. While this confirms potential high-affinity binding, it provides a limited snapshot that cannot capture the dynamic behavior, conformational flexibility, or solvation effects critical for fully understanding target engagement (98). Consequently, the theoretical affinities and binding modes reported here represent a preliminary structural hypothesis that requires further validation.

To bridge these gaps, methodologically rigorous experimental validation is warranted—for instance, employing Western blot to quantify baricitinib’s suppression of AKT1/STAT3 phosphorylation. Concurrently, prospective clinical studies in CKD patients are essential to directly evaluate renal efficacy and safety endpoints such as eGFR slope and infection incidence, thereby moving beyond the associative signals derived from pharmacovigilance. To address the aforementioned limitations, future work should first aim to contextualize the current systemic findings within the kidney. This involves integrating kidney-specific omics data: Single-cell transcriptomics from human CKD biopsies can both refine the existing target network into cell-type-specific signatures and enable the discovery of novel targets, while spatial proteomics can validate target expression within pathological renal niches (99, 100). As a crucial computational next step, the docked complexes predicted herein must be validated through molecular dynamics simulations. These simulations are indispensable to verify the temporal stability of the interactions, to quantify binding free energies with greater rigor by accounting for solvation and flexibility, and to elucidate detailed interaction networks and potential allosteric mechanisms—thereby transforming our static structural hypotheses into a dynamic and mechanistically coherent profile of baricitinib’s polypharmacology in CKD (101, 102).

This refined computational profile establishes a strong prior for direct experimental target validation. To empirically confirm the binding events central to our polypharmacology hypothesis, a logical next step employs biophysical techniques such as surface plasmon resonance (SPR) to quantify the affinity of baricitinib for purified core targets like SRC and AKT1, providing a decisive experimental benchmark for the docking predictions (103, 104). Subsequent cellular target engagement can be assessed by methods like the cellular thermal shift assay (CETSA) in relevant renal cells (105). Crucially, beyond confirming inhibition of the core hubs, a comprehensive kinase activity profiling using high-throughput platforms could systematically map baricitinib’s selectivity across the kinome, quantifying its potential off-target effects and further refining the polypharmacology network (106). Furthermore, co-immunoprecipitation (Co-IP) could examine whether baricitinib disrupts specific, disease-relevant protein complexes—such as those within the JAK-STAT axis or those nucleated by EGFR (107). This targeted *in vitro* framework is designed to furnish mechanistic proof of principle before advancing to phenotypic animal models.

Following this computational refinement, the predictive insights define a critical next step: the experimental validation of the polypharmacology hypothesis in mechanistically informative preclinical models. Studies in models such as 5/6 Nx model or adenine-induced nephropathy should directly test whether the renoprotective effects of baricitinib depend on its broader target engagement (108, 109). A definitive approach would employ targeted perturbation of key network hubs, for instance by assessing the drug’s efficacy under concurrent SRC inhibition or in combination with AKT1 or EGFR pathway modulation (110, 111). Such preclinical studies move beyond phenotypic confirmation to test the functional necessity of the predicted multi-target axis, a process essential to substantiate the systems pharmacology proposed in this study (112). Ultimately, the hypotheses generated here require validation through experimental models of kidney disease and prospective clinical trials.

Baricitinib presents a “double-edged sword” in CKD management, in that its multi-target synergism may delay fibrosis progression, yet concurrently entails dose-dependent risks of immunosuppression and thrombosis. Clinically, stratified dosing protocols and dynamic monitoring parameters grounded in target affinity data, such as docking scores, and known pharmacokinetic properties from the literature offer quantifiable decision-making support for personalized therapy. The organ-specificity of target functions further implies potential cross-disease application value.

## 5 Conclusion

By integrating computational network toxicology forecasts, molecular docking results, and real-world pharmacovigilance evidence from the drug’s broader clinical use, this study provides a systematic, hypothesis-generating exploration of baricitinib’s potential profile in CKD. Our analysis suggested that baricitinib may synergistically disrupts JAK-STAT/MAPK/PI3K-Akt inflammatory cascades and TGF-β1/Smad3 fibrogenic signaling through predicted interactions of key regulatory targets, namely AKT1, EGFR, ESR1, SRC, and STAT3, for which it shows higher predicted binding affinity than for its canonical target JAK1. This putative multi-target mechanism offers a mechanistic hypothesis for its potential to ameliorate renal pathology. While real-world safety data from its broader clinical use highlight risks of infection and thrombosis, no disproportionate renal adverse signal was detected. These pharmacovigilance findings, combined with our computational predictions, generate a testable hypothesis that baricitinib may exert renoprotective effects via multi-target modulation in CKD, though this remains speculative and requires prospective validation in CKD-specific cohorts. Consequently, this work outlines key considerations for future trial design, such as the potential need for dose adjustment in advanced renal impairment and vigilant monitoring for infections and thrombosis. Collectively, this exploratory study provides a hypothesis-generating rationale and a safety-informed framework for future research on baricitinib in CKD. It illustrates a paradigm for investigating JAK inhibitors that extends beyond single-pathway blockade toward the potential for multi-dimensional modulation of fibro-inflammatory disease processes.

## Data Availability

Publicly available datasets were analyzed in this study. This data can be found here: The pharmacovigilance analysis in this study utilized publicly accessible data from the FAERS, a spontaneous reporting database maintained by the U.S. FDA. All data were obtained directly from the official FAERS public dashboard: https://www.fda.gov/drugs/drug-approvals-and-databases/fda-adverse-event-reporting-system-faers-database.

## Footnotes

1
https://pubchem.ncbi.nlm.nih.gov/

2
https://tox.charite.de/protox3/

3
https://admetmesh.scbdd.com

4
https://www.ebi.ac.uk/chembl/

5
http://stitch.embl.de/

6
http://www.swisstargetprediction.ch/

7
https://www.uniprot.org/

8
https://www.genecards.org/

9
https://www.omim.org/

10
https://db.idrblab.net/ttd/

11
https://cn.string-db.org/

12
https://www.rcsb.org/

13
https://cadd.labshare.cn/cb-dock2/
