# Supplementary material for: Baricitinib in chronic kidney disease: an exploratory analysis integrating network toxicology, molecular docking and pharmacovigilance
Source: Front Med (Lausanne). 2026 Jan 9;12:1717739. doi: 10.3389/fmed.2025.1717739 (PMC12827112; doi:10.3389/fmed.2025.1717739)
Supplement: Supplementary file 1 [file Table_1.docx]

Supplementary Table S1. Complete list of the top 30 signal strength of AEs of baricitinib at the PTs level in FAERS database.

| SOC |  | PTs |  | Case Reports |  | ROR  (95% CI) |  | PRR  (95% CI) |
| --- | --- | --- | --- | --- | --- | --- | --- | --- |
| Infections and infestations |  | Herpes zoster meningitis |  | 7 |  | 153.36  (71.34, 329.67) |  | 153.27  (71.36, 329.18) |
| Infections and infestations |  | Diverticulitis intestinal perforated |  | 12 |  | 131.5  (73.48, 235.35) |  | 131.38  (72.97, 236.53) |
| Infections and infestations |  | Eczema herpeticum |  | 9 |  | 75.3  (38.76, 146.3) |  | 75.24  (38.64, 146.51) |
| Infections and infestations |  | Spinal cord abscess |  | 5 |  | 66.86  (27.47,162.71) |  | 66.83  (27.66, 161.44) |
| Infections and infestations |  | Herpes zoster disseminated |  | 9 |  | 61.08  (31.5, 118.44) |  | 61.04  (31.35, 118.86) |
| Infections and infestations |  | Pneumonia staphylococcal |  | 15 |  | 52.08  (31.21, 86.9) |  | 52.02  (31.25, 86.59) |
| Infections and infestations |  | Lymph node tuberculosis |  | 5 |  | 40.78  (16.84, 98.76) |  | 40.76  (16.87, 98.47) |
| Infections and infestations |  | Herpes zoster meningoencephalitis |  | 4 |  | 39.65  (14.76, 106.56) |  | 39.64  (14.88, 105.62) |
| Infections and infestations |  | Endocarditis staphylococcal |  | 3 |  | 37.91  (12.11, 118.65) |  | 37.9  (12.16, 118.13) |
| Infections and infestations |  | Ophthalmic herpes zoster |  | 16 |  | 34.78  (21.22, 57) |  | 34.74  (21.28, 56.71) |
| investigations |  | sputum culture positive |  | 21 |  | 234.77  (149.8, 367.95) |  | 234.38  (149.33, 367.88) |
| investigations |  | stenotrophomonas test positive |  | 4 |  | 204.43  (73.51, 568.51) |  | 204.37  (73.75, 566.3) |
| investigations |  | aspergillus test positive |  | 4 |  | 64.33  (23.81, 173.78) |  | 64.31  (23.67, 174.74) |
| investigations |  | blood culture positive |  | 15 |  | 60.36  (36.14, 100.82) |  | 60.29  (36.22, 100.36) |
| investigations |  | klebsiella test positive |  | 7 |  | 59.86  (28.26, 126.8) |  | 59.83  (28.41, 126) |
| investigations |  | candida test positive |  | 3 |  | 53.07  (16.89, 166.72) |  | 53.06  (17.02, 165.38) |
| investigations |  | fibrin d dimer increased |  | 34 |  | 47.56  (33.85, 66.82) |  | 47.43  (33.99, 66.19) |
| investigations |  | enterococcus test positive |  | 4 |  | 36.95  (13.76, 99.23) |  | 36.93  (13.86, 98.4) |
| neoplasms benign, malignant  and unspecified  (incl cysts and polyps) |  | diffuse large b-cell lymphoma stage iv |  | 4 |  | 55.09  (20.43, 148.52) |  | 55.07  (20.27, 149.64) |
| neoplasms benign, malignant  and unspecified  (incl cysts and polyps) |  | essential thrombocythaemia |  | 3 |  | 38.12  (12.18, 119.31) |  | 38.11  (12.23, 118.78) |
| neoplasms benign, malignant  and unspecified  (incl cysts and polyps) |  | lentigo maligna |  | 3 |  | 36.31  (11.61, 113.61) |  | 36.3  (11.65, 113.14) |
| neoplasms benign, malignant  and unspecified  (incl cysts and polyps) |  | bronchial carcinoma |  | 4 |  | 33.57  (12.51, 90.11) |  | 33.56  (12.6, 89.42) |
| neoplasms benign, malignant  and unspecified  (incl cysts and polyps) |  | adenocarcinoma gastric |  | 7 |  | 32.53  (15.42, 68.61) |  | 32.51  (15.44, 68.47) |
| nervous system disorders |  | cerebral artery thrombosis |  | 4 |  | 45.1  (16.76, 121.33) |  | 45.08  (16.92, 120.11) |
| nervous system disorders |  | post herpetic neuralgia |  | 10 |  | 34.7  (18.58, 64.82) |  | 34.68  (18.52, 64.93) |
| nervous system disorders |  | thrombotic cerebral infarction |  | 3 |  | 34.15  (10.92, 106.8) |  | 34.15  (10.96, 106.44) |
| respiratory, thoracic  and mediastinal disorders |  | pulmonary infarction |  | 10 |  | 41.99  (22.46, 78.5) |  | 41.95  (22.4, 78.55) |
| respiratory, thoracic  and mediastinal disorders |  | pulmonary artery thrombosis |  | 5 |  | 37.22  (15.38, 90.07) |  | 37.2  (15.4, 89.87) |
| skin and subcutaneous tissue disorders |  | alopecia areata |  | 30 |  | 62.8  (43.68, 90.29) |  | 62.65  (44.03, 89.15) |
| gastrointestinal disorders |  | diverticular perforation |  | 13 |  | 41.27  (23.84, 71.44) |  | 41.23  (23.82, 71.38) |
